# Spy'ing on differentiation in neuroblastoma

**DOI:** 10.18632/oncotarget.2325

**Published:** 2014-08-07

**Authors:** Monika Podkowa, Meredith S. Irwin

**Affiliations:** Hospital for Sick Children Research Institute, Cell Biology Program

Neuroblastoma (NB) is a highly malignant pediatric tumor derived from primordial neural crest cells that give rise to sympathetic neural ganglia and adrenal medulla. Most tumors are located in the adrenal gland or along the sympathetic chain in the neck, chest, abdomen or pelvis. More than half of patients have metastases and <50% survive. NB is a heterogeneous disease, ranging from tumors with poorly differentiated neuroblasts to those consisting of fully differentiated sympathetic neurons, and the degree of differentiation has prognostic significance [1]. Furthermore, the clinical presentation ranges from spontaneous regression, most commonly seen in infants and associated with maturation or differentiation, to aggressive metastatic tumors that are more undifferentiated and resistant to chemotherapy. Thus, there has been great interest in differentiation therapeutic strategies. The retinoid isotretinoin (cis-retinoic acid)-induces NB differentiation and cell cycle arrest *in vitro* and is currently used in treatment regimens for NB patients [1].

**Figure 1:**
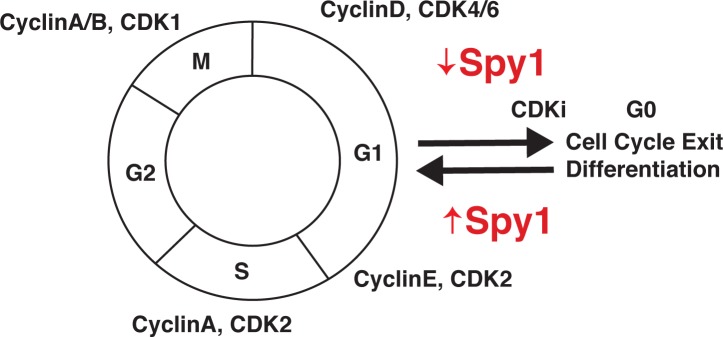
Spy1 is a novel atypical Cyclin regualing NB cell cycle progression A simplified version of cell cycle progression and its regulators. Spy1 levels regulate NB cell exit and differentiation.

Proper regulation of the cell cycle is tightly controlled by Cyclins, Cyclin dependent kinases (CDKs) and CDK inhibitors (CDKis) (Fig 1). Tumor cells can arise from progenitor cells that fail to exit the cell cycle and differentiate, or from de-differentiated cells that have re-entered the cell cycle. Cyclins, CDKs and CDKis are often deregulated in cancer. A highly conserved family of “Cyclin-like” proteins called the Speedy/RINGO family are CDK binding partners that control orderly progression through the cell cycle. The originally characterized member, Spy1, is required for cell cycle re-entry and unlike previously described classical Cyclin proteins, bypasses conventional inhibitory mechanisms to control CDK2 activity and G1-S phase transition [2]. The Spy1-CDK2 complex does not rely on CDK2 phosphorylation by CDK activating kinase (CAK) and is less sensitive to inhibitory phosphorylation by regulators such as p21Cip1 and p27Kip1. Thus, Spy1 is able to override cell cycle checkpoints, and allows for small pools of CDKs to be active while still globally restricting CDK activity. Spy1 has been shown to be upregulated in a number of human cancers including gliomas, where expression levels are increased in higher grade tumors, and amplification of the Spy1 encoding *SPDYA* gene and overexpression of the Spy1 effector CDK2 correlate with poor survival [3]. Interestingly, Spy1 was shown to regulate the “stemness” properties and differentiation of central nervous system (CNS) brain tumor initiating cell (BTIC) populations.

In May issue of *Oncoscience*, Porter and colleagues characterize the roles of Spy1 in proliferation, differentiation and self-renewal of the peripheral nervous system tumor NB [4]. In contrast to CNS tumors, NB cells that have enhanced tumor initiating cell (TIC) properties *in vivo* cannot be identified with one single marker [5]. However, similar to previous NB studies, Lubanska and colleagues utilize CD133+ and CD133- NB cells to model NB cells with “stem like properties”. They demonstrate that Spy1 protein levels are downregulated during retionoic acid-induced differentiation, while overexpression of Spy1 increases proliferation and suppresses differentiation. Interestingly, Spy1 levels were significantly elevated in cells cultured as neurospheres compared to monolayers. Spy1 overexpressing NB cells demonstrated increased self-renewal in a neurosphere formation assays and expressed markers indicative of multipotency (*Oct-4*, *BMI1*, *OLIG2*, *GFAP* and *CD133*). ShRNA-mediated downregulation of Spy1 resulted in decreased NB cell proliferation and decreased levels of CD133 expression. Furthermore, Spy1 overexpression in CD133- NB cells resulted in increased neurosphere formation and levels of CD133, c-MYC and Ki67, while Spy1 knockdown in CD133+ NB cells resulted in significant decline in the number of spheres formed and decreased levels of Ki67 and c-MYC. Thus, Spy1, the atypical regulator of cell cycle progression suppresses differentiation, drives proliferation and stem-like characteristics in NB, particularly in the CD133+ populations. These findings suggest that Spy1 may have oncogenic properties and anti-proliferative effects in the *MYCN*-non amplified SH-SY5Y cell line, perhaps selectively in cells with TIC properties. Although Spy1 expression in NB tumors has not been reported, *SPYDA* maps to 2p23.2, a region that in some NB may be co-amplified with *MYCN* (2p24.3). It will be interesting to determine whether Spy1 protein is upregulated in NB tumors and whether levels correlate with differentiation, *MYCN* status or other prognostic factors.

Cell cycle aberrations involving G1-regulating genes have been identified in tumors, including NB where copy number gains and overexpression of CDK4/6 and Cyclin D have been detected [6]. Furthermore, many reports have indicated that CDK activities decline during differentiation, and inhibition of G1 regulating genes, CDK4 or Cyclin D1, has been shown to induce NB cell differentiation. In addition, suppression of the activity of the Spy1 effector CDK2 is synthetically lethal in *MYCN* amplified NB cells [7]. In light of these aberrations in CDK/Cyclin functions, the novel role of Spy1 in regulating NB differentiation, proliferation and stem-like characteristics is particularly intriguing and may provide rationale for targeting Spy1 and its effectors and regulators (*eg* CDK2) to induce cell cycle arrest and differentiation. Additional mechanistic studies may lend insight into biomarkers in NB tumors, such as *MYCN* amplification or Spy1 upregulation to predict sensitivity to agents targeting Spy1/CDK2. Taken together, the findings implicating the atypical Cyclin Spy1 in differentiation of gliomas and NB suggest that this pathway may be exploited as a therapeutic target in these neuronal tumors, possibly by targeting the TIC-like cells to promote differentiation and cell cycle exit.
